# A Comparison of Carbon Footprint and Production Cost of Different Pasta Products Based on Whole Egg and Pea Flour

**DOI:** 10.3390/foods5010017

**Published:** 2016-03-04

**Authors:** Antonia Nette, Patricia Wolf, Oliver Schlüter, Andreas Meyer-Aurich

**Affiliations:** Department Technology Assessment and Substance Flows, Leibniz-Institute for Agricultural Engineering Potsdam-Bornim, Max-Eyth-Allee 100, 14469 Potsdam, Germany; anette@atb-potsdam.de (A.N.); pwolf@atb-potsdam.de (P.W.); oschlueter@atb-potsdam.de (O.S.)

**Keywords:** protein, pea flour, carbon footprint, LCA, pasta

## Abstract

Feed and food production are *inter alia* reasons for high greenhouse gas emissions. Greenhouse gas emissions could be reduced by the replacement of animal components with plant components in processed food products, such as pasta. The main components currently used for pasta are semolina, and water, as well as additional egg. The hypothesis of this paper is that the substitution of whole egg with plant-based ingredients, for example from peas, in such a product might lead to reduced greenhouse gas emissions (GHG) and thus a reduced carbon footprint at economically reasonable costs. The costs and carbon footprints of two pasta types, produced with egg or pea protein, are calculated. Plant protein–based pasta products proved to cause 0.57 kg CO_2_ equivalents (CO_2_eq) (31%) per kg pasta less greenhouse gas emissions than animal-based pasta, while the cost of production increases by 10% to 3.00 €/kg pasta.

## 1. Introduction

Feed and food production contribute substantially to the emissions of greenhouse gases, which are known to cause global warming with serious environmental and economic threads [1]. The relevant greenhouse gas fluxes affected by agronomic activities are the fluxes of carbon dioxide (CO_2_), methane (CH_4_) and nitrous oxide (N_2_O). In particular, livestock, causing 18% of the global greenhouse gas emissions, has a major share [2,3]. Subsequently, food with animal protein components, such as dairy (cheese: 8.8 kg CO_2_eq/kg cheese) and meat products (beef: 29.0 kg CO_2_eq/kg beef), show high greenhouse gas emissions. Besides animal products, few vegetables and cereals (tomato: 5.3 kg CO_2_eq/kg tomato; rice: 1.2 kg CO_2_eq/kg rice) are also generating high emissions. Greenhouse gas emissions could be reduced by the replacement of animal with plant components in foods. It is conceivable to use grain legumes, such as peas or beans, as such plant replacement components. Grain legumes, such as peas with a carbon footprint of 0.49 kg CO_2_eq/kg pea, have been suggested as a very efficient source of protein in terms of greenhouse gas (GHG) emissions per kg [4]. Pea-based protein has proved to be very well suited for the fortification of pasta products and the improvement of techno-functional and sensorial properties, and thus could very well substitute animal-based ingredients of processed foods [5].

Besides the greenhouse gas mitigation effect of substituting animal protein with legume protein, the integration of grain legumes in crop rotations has positive effects on soil fertility and soil health [6]. Owing to the formation of taproots, grain legumes improve the soil structure and result in a more diverse crop rotation. Furthermore, the plants form a symbiosis with bacteria of the family Rhizobiaceae, which can bind atmospheric nitrogen in the soil and make it available for plants [6,7]. An enrichment of grain legumes such as peas in foodstuffs has, in addition to the aforementioned improvements in agriculture, positive effects on human health. They are rich in certain minerals and vitamins [8]. The content of crude protein of 225 g per kg dry matter [9] qualifies peas to be used as replacers for animal-derived proteins, contributing to enhancing the protein content of cereal-based meals and to improving the nutritional status of cereal-based diets. Regarding amino acid composition, especially combinations of cereal and legume proteins are beneficial. As cereal proteins are deficient in certain essential amino acids, particularly lysine [8], legumes have been reported to contain adequate amounts of lysine (15.7 g/kg of dry matter) [9]. Thus, thanks to their inherent botanical make-up and the basis of the ingredients, grain legumes can increase the amount of protein in cereal-based diets [8,9]. In addition, high amounts of protein, a low glycemic index and high fiber content are other favorable factors for biological activities which are essential to human health. It has been reported that grain legumes reduce the risk of cardiovascular diseases, diabetes or cancer, especially colon cancer [10,11,12], which may also be a reason for the increased interest in using pea-based ingredients in processed foods.

In contrast to the aforementioned health benefits, the consumption of proteins from grain legumes compared to animal proteins is very low. This is *inter alia* due to a poor digestibility of legumes and the presence of anti-nutritional substances in the plant [13,14]. In order to make better use of legumes in processed foods, it is necessary to eliminate the aforementioned restrictive factors and to communicate the benefits to the consumer. However, due to several ethical and personal reasons, nowadays, more consumers are interested in replacing proteins of animal origin.

One possible food product in which ingredients of animal origin could be replaced by plant components is pasta. The currently used main components of pasta are semolina (made of wheat) and water. Furthermore, eggs in the form of raw or pasteurized whole egg can also be added [6,15,16,17,18]. This paper analyzes the use of two different pasta products, with and without animal ingredients, which differ in costs and product-specific GHG emissions.

## 2. Materials and Methods

### 2.1. Modeling the Value Chain of Two Pasta Products Based on Protein from Egg and Peas

**Figure 1:**
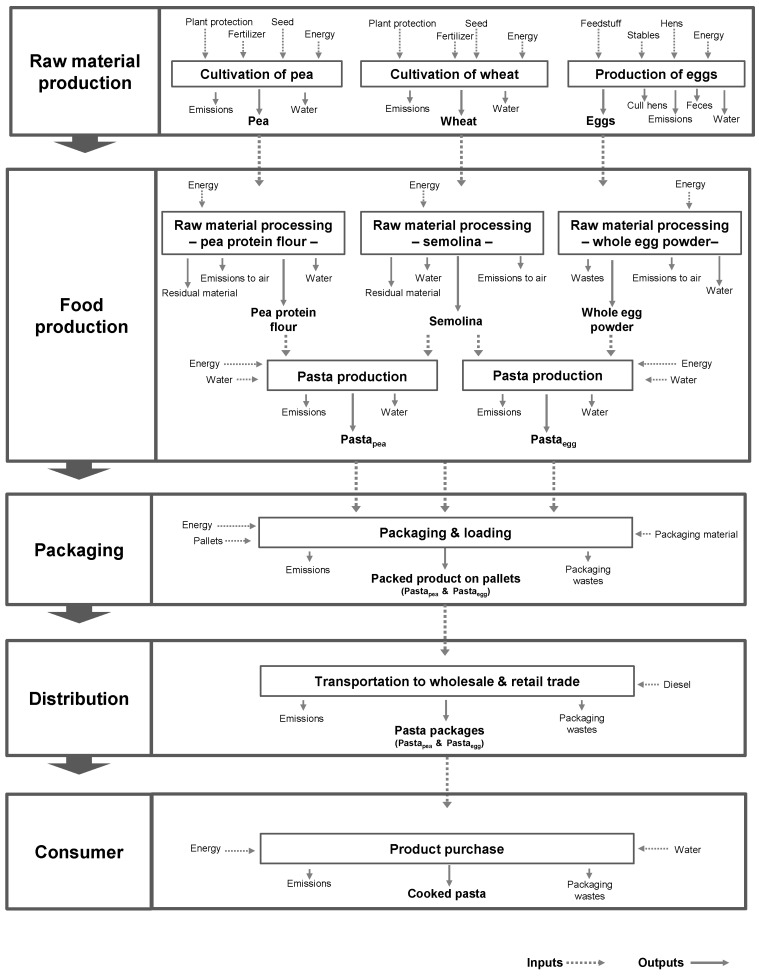
The value chain of the pasta production for Pasta_egg_ and Pasta_pea_.

For the environmental and economic analyses, the first step is to analyze the value chain of pasta production. A typical value chain of pasta production consists of the main process steps: “raw material production”, “food production”, “packaging”, “distribution” and “consumer”. This includes the corresponding sub-processes (Figure 1).

Typically, for one kg of pasta enhanced with protein from hens’ eggs (Pasta_egg_), 0.8 kg semolina, 0.2 kg whole egg powder and 0.3 L water are needed [17,19]. For comparison, we calculated costs and greenhouse gas emissions of a pasta product (Pasta_pea_) which consists of 0.2 kg pea protein flour instead of whole egg powder, based on data provided by Nielsen *et al.* [5].

Input and output data for the main process step of “raw material production” were taken from available databases on agricultural production [20,21,22,23,24]. The production data of pea protein flour are given by the company “GEA Westfalia Seperator Group” [25] and available data from scientific literature [26,27]. Data on spray-dried whole egg powder and on the whole process of dry pasta production (pasta production, packaging, distribution and consumer) were gathered from available data from pasta producers and scientific literature [17,19,24,28,29].

### 2.2. Cost Analysis of the Pasta Products Based on Protein from Egg and Peas

For the cost analysis, costs of the ingredients and the production, as well as the sale price for the wholesale and retail trade, were gathered from available data sources. The purchase prices of the ingredients, semolina (0.50 €/kg dry matter), whole egg powder (1.90 €/kg dry matter) and pea protein flour (2.50 €/kg dry matter), were based on the expert judgment of a project partner [30]. A water price of 2.60 €/m³ was assumed [31]. During processing, 0.3 L of water per kg pasta was accounted for, which in part was removed with the drying to a water content of 12.5% for both pastas [19].

According to Panno *et al.* [32], the costs of the process step of “pasta production” consist of the costs of raw materials (77%), labor (14%), electric and thermal energy (6%) as well as packaging (3%). For the Pasta_pea_, the calculated values for labor, electricity and thermal energy of the Pasta_egg_ were used. The price calculations for the wholesale and retail trade are determined by the procurement costs, the handling costs and the mark-up, according to calculation templates of the Federal Ministry for Economic Affairs and Energy [33]. Reference costs include the costs for shipping or the delivery of products. The handling costs include, for example, the costs for administration or sale negotiations. For the calculation of the wholesale price and retail trade for Pasta_egg_ and Pasta_pea_, handling costs of 35% and a mark-up of 10% were used.

### 2.3. Carbon Footprint of the Pasta Products Based on Protein from Egg and Peas

The carbon footprints of the two pasta products were based on the estimated fluxes of all relevant GHGs, mainly CO_2_, CH_4_ and N_2_O, according to their global warming potential for a 100-year time frame [34] and expressed in CO_2_ equivalents (CO_2_eq) per kg pasta product, based on a life cycle assessment approach. Accordingly, CO_2_eq emissions from the production taking into account all relevant pre-chain emissions were estimated for the carbon footprint of the pasta products according to the relevant products and processes involved in the different value chains (Table 1). The GHG emissions of the production of wheat, pea, egg and water were downscaled to the amounts correspondingly required for the pasta product [4]. These were 0.8 kg semolina, 0.3 L water and 0.2 kg whole egg for the production of one kg dry Pasta_egg_ and 0.8 kg semolina, 0.3 L water and 0.2 kg pea protein flour for the production of one kg Pasta_pea_. The emissions for the whole egg powder production were calculated according to a steam-drying process, using a vibro-fluid bed dryer [35]. The process data were translated into GHG emissions for the respective whole egg powder using data provided by the Ecoinvent database [36].

For the pea protein flour production and water, data provided by the Ecoinvent database [36] were used. Data for the milling process of wheat, pasta production, packaging and distribution were taken from Ruini and Marino [37].

## 3. Results

### 3.1. Costs of Pasta Production

**Figure 2:**
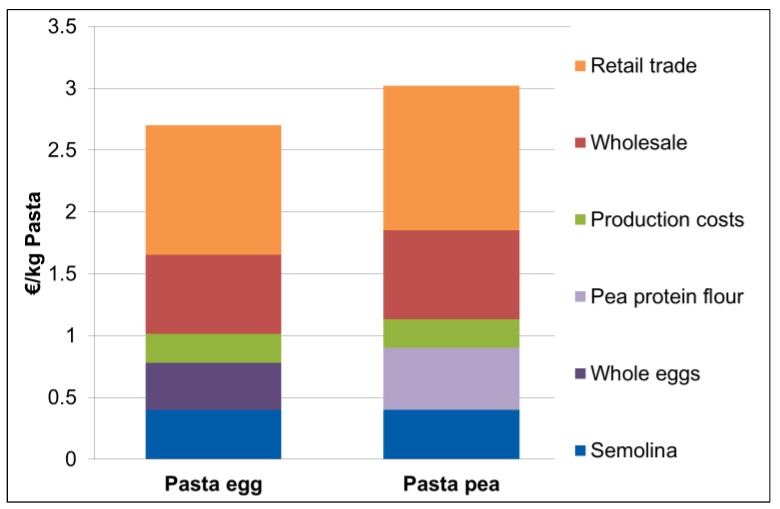
Price calculation for Pasta_egg_ and Pasta_pea_.

The total production costs for one kg Pasta_egg_ with a content of 0.8 kg semolina, 0.3 L water and 0.2 kg whole egg sum to 1.00 €/kg dry pasta (Figure 2). In comparison, production costs for one kg Pasta_pea_ with 0.2 kg pea protein flour instead of whole egg sum to 1.10 €/kg dry pasta. Including assumptions on the wholesale process results in prices of 1.65 €/kg pasta Pasta_egg_ and 1.85 €/kg pasta for Pasta_pea_. The calculated retail price which has to be paid by the consumer is 2.70 €/kg for the Pasta_egg_ and 3.00 €/kg pasta for the Pasta_pea_. Thus, the Pasta_egg_ has an estimated 0.30 €/kg pasta lower price than the Pasta_pea_.

Decisive factors for the retail price are the costs of ingredients and the production process. The pea protein flour has a purchase price of 2.50 €/kg dry matter more expensive than the whole egg (purchase price of 1.90 €/kg dry matter). Accordingly, the Pasta_egg_ has a higher retail price.

### 3.2. Carbon Footprint of Pasta Production

**Figure 3:**
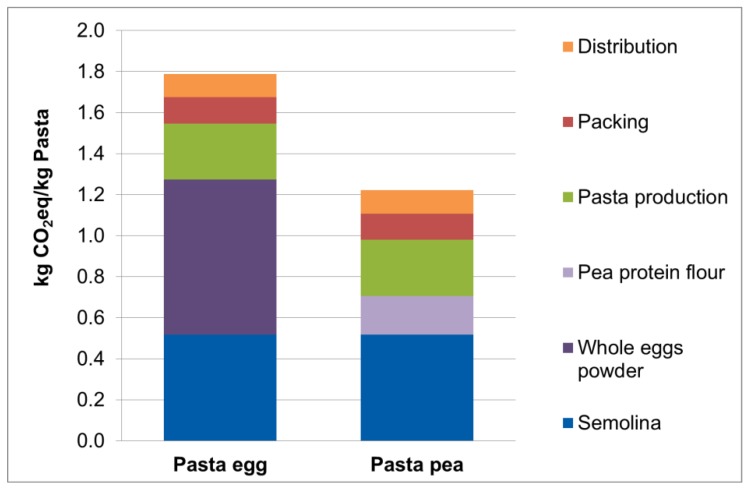
Carbon footprint of different pasta types.

The CO_2_eq emissions for the whole production of the two pasta types (from the agricultural steps to the final product) are 1.79 kg CO_2_eq/kg dry pasta for the Pasta_egg_ and 1.22 kg CO_2_eq/kg dry pasta for the Pasta_pea_ (Figure 3).

The difference between the emissions stems from the high emissions of the whole egg powder production. The production of the pea protein flour requires a high amount of energy for the processes of grinding as well as air classification. Anyhow, in comparison to the egg production, the total emissions of pea flour production are much lower than the production of the whole egg powder.

## 4. Discussion

The production costs of the two pasta products differ a little (0.30 €/kg pasta), and this is mainly determined by the higher costs of pea protein flour extraction compared to the costs of egg pasteurization. This finding is in accordance with others [24,27]. In the case of pea protein flour, one possible reason for the higher protein price may be the limited demand of pea protein flour compared to whole egg. However, the protein concentration of the pasta based on whole egg is slightly lower (204 g/kg dry pasta) compared to the pasta based on pea flour (212 g/kg dry pasta). Taking this difference into account with a protein-corrected composition of the two pastas, however, only marginally affects the cost difference of the two pastas (0.29 €/kg pasta).

The techno-functional and sensory quality of a new product is also an important criterion for the consumer. The aspects of sensory attributes such as the taste or the overall impression of a pea-rich pasta product were analyzed by Linsberger *et al.* [16]. The pasta products of their study consisted of 50% pea flour and 50% durum flour or 100% pea flour. With regard to the techno-functionality, it was found that an increase of legumes causes a higher cooking loss. Furthermore, the taste, structure and color of legume-rich pasta products were judged inferior, especially for durum flour pasta. A reduction of the legume flour to 20% pea protein flour in the product has been proven to achieve much better results in such a sensorial test [5].

In comparison to the reference pasta product (Pasta_egg_), Pasta_pea_ was shown to emit 35.5% (0.42 kg CO_2_eq/kg product) less GHGs over the whole value chain. The difference between the emissions stems from the high emissions of whole egg powder production and the pre-chain emissions due to feed and husbandry of the hens. The production of the pea protein flour also requires a high amount of energy for the processes of grinding as well as air classification, resulting in high GHG emissions. However, in comparison to the egg production, the total emissions of pea-based pasta are still low [15,27]. This finding is in accordance with other studies, which show the potential impact of changed diets on the environment and especially on greenhouse gas emissions [4,38,39].

Compared to the small change in the ingredients of the product, the impact on the carbon footprint is substantial. Mainly due to higher costs of the pea protein flour, the calculated selling price would be increased 10% (0.30 €/kg pasta). It can be imagined that a communication of the impact on the carbon footprint to the consumer could be a strong selling argument, which justifies the higher price. Furthermore, the positive effects of pea cultivation for agriculture, and the likewise positive effects for human health, may be a benefit for the consumer and society as a whole.

## Figures and Tables

**Table 1:** Relevant greenhouse gas (GHG) emission fluxes for the products and processes of the considered value chains.

	Reference Unit	GHG Emissions (kg CO_2eq_^1^)	Source
**Products**			
Pea protein flour	kg protein	0.94	[36]
Wheat	kg wheat	0.58	[4]
Egg	kg egg	3.00	[4]
Water	kg water	3.19 × 10^-4^	[36]
**Processes**			
Egg drying	kg egg	0.78	[35]
Semolina milling	kg wheat	0.06	[37]
Pasta production	kg pasta	0.27	[37]
Packaging	kg pasta	0.13	[37]
Transport and Distribution	kg pasta	0.11	[37]

^1^ CO_2_ equivalents.
